# Pyriproxyfen, a juvenile hormone analog, damages midgut cells and interferes with behaviors of *Aedes aegypti* larvae

**DOI:** 10.7717/peerj.7489

**Published:** 2019-09-04

**Authors:** Muhammad Fiaz, Luis Carlos Martínez, Angelica Plata-Rueda, Wagner Gonzaga Gonçalves, Debora Linhares Lino de Souza, Jamile Fernanda Silva Cossolin, Paulo Eduardo Gomes Rodrigues Carvalho, Gustavo Ferreira Martins, José Eduardo Serrão

**Affiliations:** 1 Departamento de Entomologia, Universidade Federal de Viçosa, Viçosa, Minas Gerais, Brazil; 2 Departamento de Biologia Geral, Universidade Federal de Viçosa, Viçosa, Minas Gerais, Brazil

**Keywords:** Autophagy, *Aedes aegypti*, Ultrastructure, Juvenile Hormone, Locomotory behavior

## Abstract

Juvenile hormone analogs (JHA) are known to interfere with growth and biosynthesis of insects with potential for insecticide action. However, there has been comparatively few data on morphological effects of JHA on insect organs. To determine pyriproxyfen effects on *Aedes aegypti* larvae, we conducted toxicity, behavioral bioassays and assessed ultrastructural effects of pyriproxyfen on midgut cells. *A. aegypti* larvae were exposed in aqueous solution of pyriproxyfen LC_50_ concentrations and evaluated for 24 h. This study fulfilled the toxic prevalence of pyriproxyfen to *A. aegypti* larvae (LC_50_ = 8.2 mg L^−1^). Behavioral observations confirmed that pyriproxyfen treatment significantly changes swimming behavior of larvae, limiting its displacement and speed. The pyriproxyfen causes remarkable histopathological and cytotoxic alterations in the midgut of larvae. Histopathological study reveals presence of cytoplasmic vacuolization and damage to brush border of the digestive cells. The main salient lesions of cytotoxic effects are occurrence of cell debris released into the midgut lumen, cytoplasm rich in lipid droplets, autophagosomes, disorganized microvilli and deformed mitochondria. Data suggest that pyriproxyfen can be used to help to control and eradicate this insect vector.

## Introduction

Among the main pathogen vectors in tropical regions, insects share a major part, causing serious diseases resulting in high mortality and economic loss (Sachs & Malaney, 2002; Shepard et al., 2011). Mosquitoes are major public health threats which transmit deadly and debilitating diseases throughout the world. Container-inhabiting mosquitoes, particularly *Aedes aegypti* Linnaeus (Diptera: Culicidae), are considered the most important vector of viral diseases in humans, including dengue fever (Jansen & Beebe, 2010; Rosen et al., 1983), urban yellow fever (Reiter, 2010), Chikungunya (Burt et al., 2012; Paupy et al., 2010) and more recently Zika virus (ZIKV) (Gardner, Chen & Sarkar, 2016; Marcondes & Ximenes, 2016; Thangamani et al., 2016). The lifespan of *Aedes aegypti* can range from two weeks to a month (Maricopa County Environmental Services, 2006), whereas larvae pass through four instars with a short time in first two, and up to 3 days in last instars (Foster & Walker, 2002). The above-mentioned diseases are increasingly becoming a global health concern, due to widespread distribution of their vectors, rapid geographical spread and high disease burden (Leta et al., 2018). During the last few years, mosquitoes have been responsible for transmitting ZIKV in Brazil and Colombia with 146,675 recognized cases (World Health Organization, 2015, 2017). There have been strong associations between existence of diseases and distribution of vectors, transmitting them (Carlson, Dougherty & Getz, 2016; Messina et al., 2016).

There is no cure for these diseases and regulating disease transmission relies mainly on vector management (Suman et al., 2018). To control this vector, immature stages must be considered a preliminary threat (Carvalho et al., 2017); this involves the use of chemical compounds which can prevent development of adult mosquitoes in aquatic environments, without damaging other organisms (Yang et al., 2017). Many insecticides are used to control *Aedes aegypti* populations, including organophosphate (Boyer et al., 2018) and pyrethroid group (World Health Organization, 2016) but their use is declining due to resistance development (Chediak et al., 2016; Goindin et al., 2017; Prophiro et al., 2011) and environmental pollutants (Eulaers et al., 2014; Jaspers et al., 2011) including air (Mäkinen et al., 2009; Yang et al., 2014), dust (Cao et al., 2014; Fang et al., 2013; Mizouchi et al., 2015), water (Cristale et al., 2013; Ding et al., 2015; Hu et al., 2014) and sediment (Chung & Ding, 2009; Cristale et al., 2013; Tan et al., 2016).

Pyriproxyfen, a juvenile hormone analog, has a unique mode of action that affects embryogenesis (Maharajan et al., 2018), metamorphosis (Barbosa et al., 2018) and reproduction of insects (Meng et al., 2018). Treatment from pyriproxyfen thus results in death typically at the pupal stage (Hustedt et al., 2017; Invest & Lucas, 2008; WHO Pesticide Evaluation Scheme (WHOPES), 2000). Additional advantage of pyriproxyfen, requiring low concentrations than other larvicides such as temephos and *Bacillus thuringiensis* var. *israelensis* (Oo et al., 2018), makes it suitable larvicide against *Aedes aegypti*. Thus, no resistance can be detected upon exposure up to 17 generations (Schaefer & Mulligan, 1991), which is a promising feature for mosquito control. Although pyriproxyfen affects metamorphosis of the insect, other insect organs may also be secondary targets (Catae et al., 2018). Among the non-target organs of the insects, the midgut has been reported to be severely damaged by xenobiotics (Gutiérrez et al., 2016; Catae et al., 2018; Fiaz et al., 2018a).

The objective of the study was to evaluate lethal and sublethal effects of pyriproxyfen against *Aedes aegypti* larvae. We investigated the toxicity, locomotory behavior, histological and ultrastructural changes of pyriproxyfen on the non-target midgut organ.

## Materials and Methods

### Insects

Late third instar (L3) *Aedes aegypti* larvae fed on cat food (Whiskas) previously, were obtained from mass rearing from the “Laboratório de Biologia Molecular de Insetos” of “Universidade Federal de Viçosa” (Viçosa, Minas Gerais, Brazil). The reason we chose late third instar larvae is to utilize early fourth instar developmental phase in our bioassays because larva spends short amount of time in the first three and up to three days in successive instar. All bioassays performed, and insect colonies were kept at 25 ± 2 °C, with a 12:12 (L:D) h photoperiod.

### Pyriproxyfen

Pyriproxyfen (TIGER^®^ 100 EC; Sumitomo Chemical Corporation, Chūō, Japan) 100 g L^−1^ was diluted in one mL water to produce a stock solution by adjusting one g L^−1^ to obtain the desired concentrations, as previously described in Fiaz et al. (2018a, 2018b).

### Toxicity test

Efficacy of pyriproxyfen was determined by calculating lethal concentrations LC_50_ under laboratory conditions. Besides control, which was distilled water, six pyriproxyfen concentrations were adjusted in one mL stock solution: 0.3125, 0.625, 1.25, 2.5, 5 and 10 µg L^−1^. From the stock solution, aliquots were obtained for each treatment and mixed with distilled water in 30 mL glass vial. Different concentrations of treatments were mixed in 25 mL of distilled water, with completely randomized design having three replications, containing 20 larvae (L3) each. Mortality was assessed every hour from the start of experiment until total mortality.

### Locomotory behavior of larvae

The behavioral recordings of *Aedes aegypti* were carried out 24 h after exposure to LC_50_ of pyriproxyfen to determine sublethal effects of treatments on larva. Bioassays were performed in a Petri dish (nine cm diameter × 1.5 cm height) with 25 mL of treatment solution diluted to the LC_50_ obtained for larvae. A single larva (L3) per petri dish was video-recorded for 10 min using a video camera (SD5 Superdynamic; modelWV-CP504; Spacecom lens 1/3″, 3–8 mm; Panasonic, Newark, NJ, USA), coupled to a computer. The measurements taken with the tracking system included distance swimmed and resting time spent in arenas. These bioassays were conducted at 25 ± 2 °C under artificial fluorescent light and each treatment had five biological replications.

### Morphological analysis of the midgut

*Aedes aegypti* larvae were exposed to LC_50_ lethal concentration of pyriproxyfen by contact and ingestion in aqueous solution for 24 h. Ten collected larvae (L3), from treatment and control were dissected in saline solution (0.1 M NaCl, 0.1 M KH_2_PO_4_, 0.1 M Na_2_HPO_4_). Dissected midgut was transferred for fixation to Zamboni’s fixative solution (Stefanini, Martino & Zamboni, 1967) and kept for 12 h at 5 °C. Dehydration of the samples were done in a graded ethanol series (70%, 80%, 90% and 95%), later embedded in historesin Leica (Leica Biosystems Nussloch GmbH, Heildelberger, Germany) and sections of three μm thickness were cut in a microtome (Leica RM2255). The acquired sections were then stained with hematoxylin and eosin and analyzed with Leica DMLS light microscope (Leica Microsystems GmbH, Wetzlar, Germany).

Another set of 10 midguts from each treatment and control *Aedes aegypti* larvae were evaluated with transmission electron microscopy and samples were fixed in 2.5% glutaraldehyde in 0.2 M sodium cacodylate buffer, pH 7.2 containing 0.2 M sucrose. Post fixation of the samples were done in 1% osmium tetroxide in the same buffer and kept for 2 h at room temperature. Samples were washed in the buffer followed by dehydration in a graded ethanol series (70%, 80%, 90% and 99%), and embedded in LR White Resin (Electron Microscopy Sciences, Fort Washington, PA, USA). Sections of 70–90 nm, obtained with glass knife in a Sorvall MT2-BMT2-B ultramicrotome (Sorvall Instruments, Wilmington, DE, USA) were stained with aqueous uranyl acetate (1%) and lead citrate (Reynolds, 1963). Those sections were then examined with Zeiss EM 109 transmission electron microscope (Carl Zeiss, Jena, Germany).

### Immunofluorescence

*Aedes aegypti* larvae (L3) were exposed to LC_50_ pyriproxyfen concentration in aqueous solution for 24 h. Midguts from treatment and control were dissected in insect physiological solution to identify cell proliferation. Five-treated midguts were used in analysis. After dissection, midgut was transferred to Zamboni’s fixative solution for 2 h, subsequently washing in 0.1M sodium phosphate buffer pH 7.2 plus 1% Tween-20 (PBST) for 2 h. The samples were incubated for 12 h with the primary antibody anti-phospho-histone H3 (1:100) in PBST, that recognize proliferating cells in the midgut of *A. aegypty* larvae (Fernandes et al., 2014, 2015), following with washing in PBST and incubation for 12 h with a FITC-conjugated anti-rabbit IgG secondary antibody (1:500). Those samples were then dehydrated in a graded ethanol series (70%, 80%, 90% and 95%) and embedded in JB4 resin. Three μm thick sections were stained with 4′,6-diamidine-2′-phenylindole dihydrochloride (DAPI) (one μg/μL) for DNA staining and then slides were analyzed and photographed under light microscope (Olympus BX-60) using a digital camera (Q-Color, 3 Olympus).

### Statistics

Lethal concentrations and their confidence limits (Finney, 1964) were subjected to probit analysis using the SAS user (v.9.0) program for Windows (SAS Institute, Cary, NC, USA). Data about behavior response were analyzed by one-way ANOVA and for mean comparisons in the bioassays Tukey’s Honestly Significant test was used at 5% significance level.

## Results

### Toxicity

**Figure 1:**
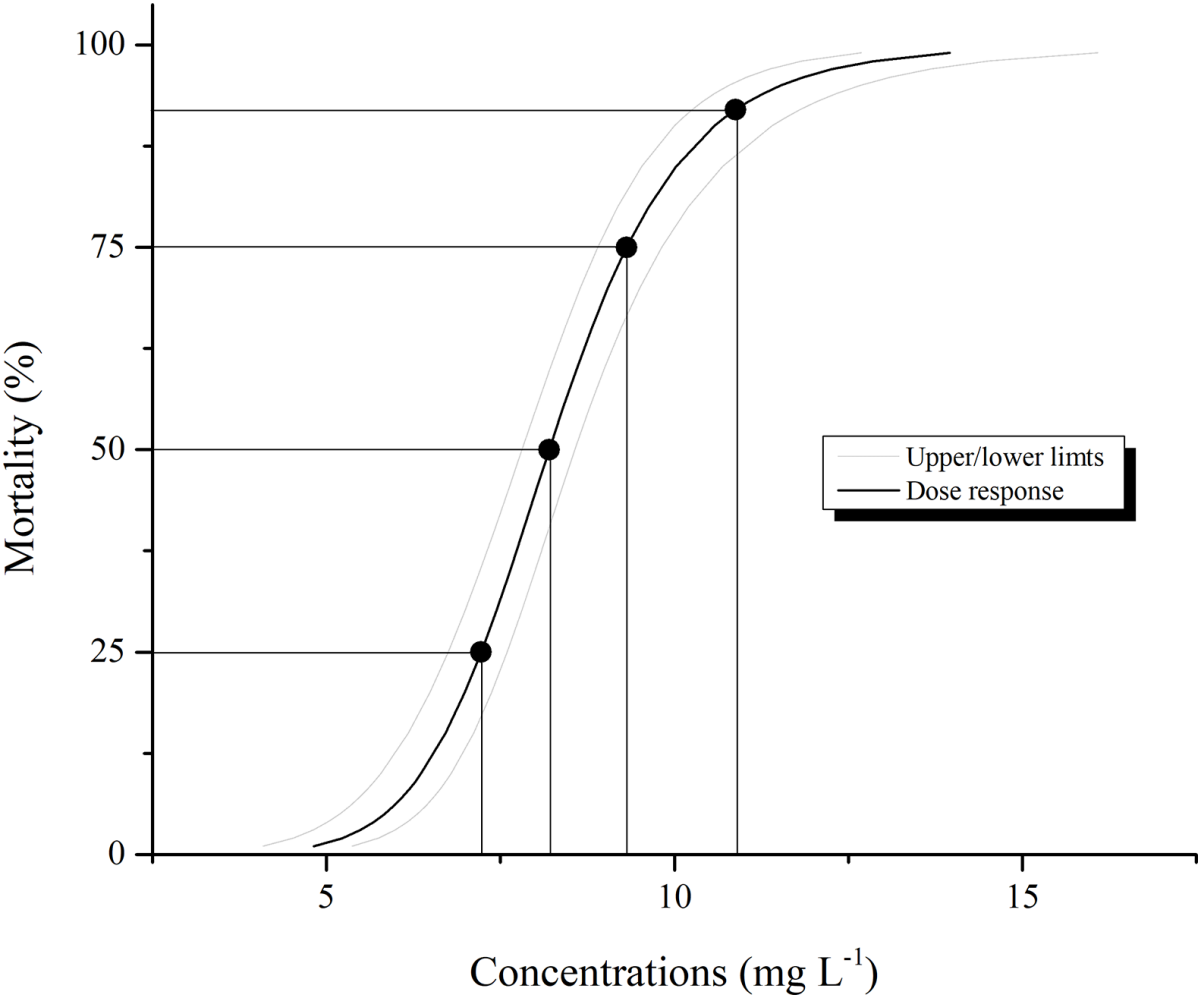
Larval mortality of *Aedes aegypti* caused by aqueous solution of pyriproxyfen. Lethal concentrations were estimated based on concentration-mortality assays using Probit analysis (χ^2^ = 20.20; d*f* = 5; *P* < 0.001). Lines denote 95% confidence intervals. Black point represents LC_25_, LC_50_, LC_75_ and LC_90_ concentrations, while LC_50_ was selected to evaluate histological, ultrastructural changes and immunofluorescence.

Larval mortality of the *Aedes aegypti* population treated with pyriproxyfen was different from that the control, as expected. Larval mortality increased with pyriproxyfen concentration in the aqueous solution with highest value obtained with 8.20 mg L^−1^ of this insecticide (Table 1). The estimated LC_50_ for pyriproxyfen obtained with the probit model was 8.20 mg L^−1^ (Fig. 1). Mortality observed in control was always <1%.

**Table 1:** Lethal pyriproxyfen concentrations to *Aedes aegypti* larvae after 24 h exposure.

Lethal Concentration (LC)	Estimated value (mg L^−1^)	Confidence limits		χ^2^
		Inferior	Superior	
LC_25_	7.22	6.75	7.60	20.20
LC_50_	8.20	7.81	8.57	
LC_75_	9.31	8.90	9.82	
LC_90_	10.5	10.0	11.4	

**Note:**

χ^2^, Chi-squared value for lethal concentrations and fiducial limits based on a log scale with significance level at *P* < 0.001.

### Locomotory behavior

**Figure 2:**
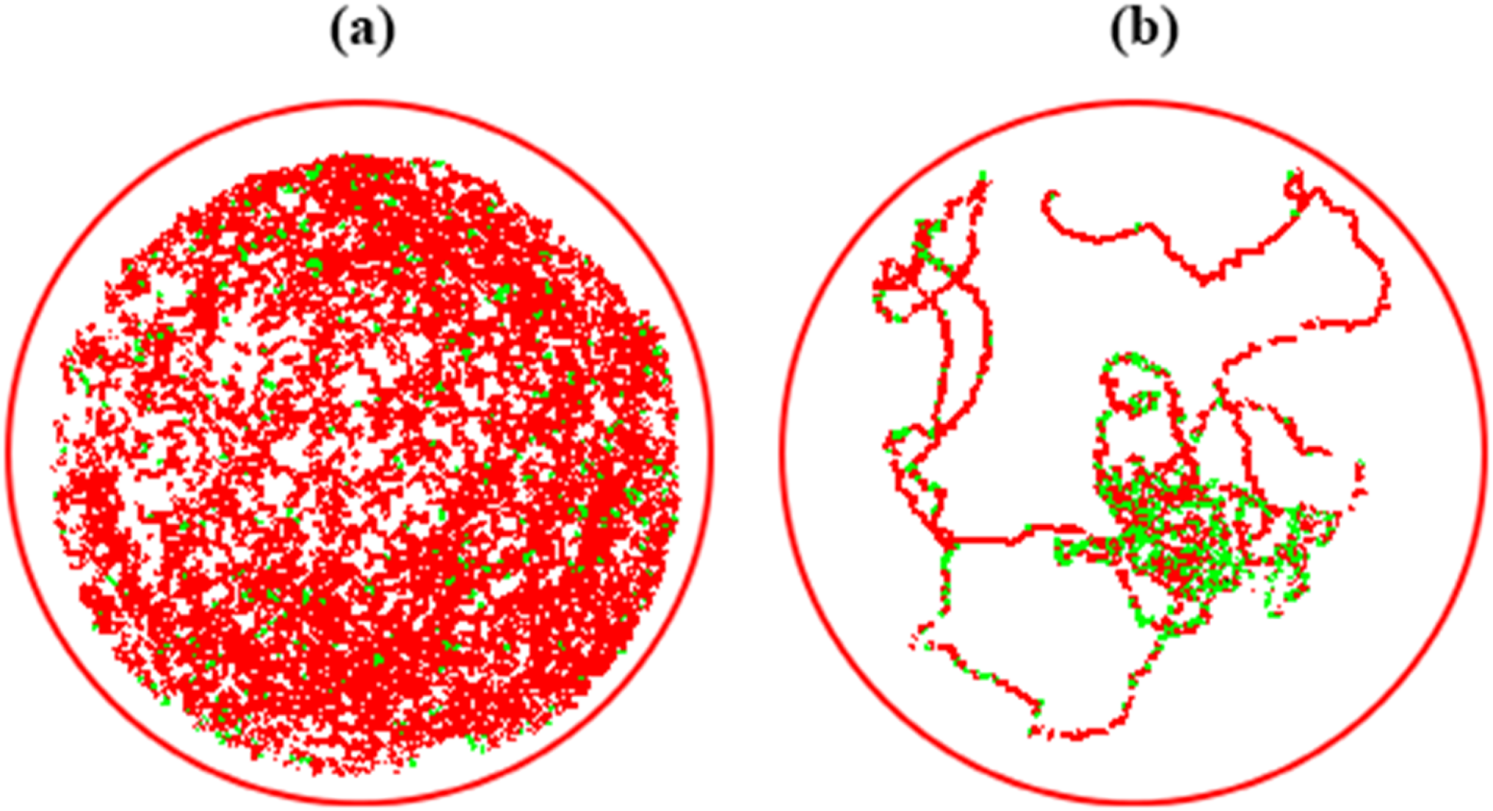
Displacement trails of *Aedes aegypti* larvae from control (A) and exposure to LC_50_ concentrations of pyriproxyfen (B). Red tracks indicate high swimming speed; green tracks indicate low (initial) velocity.

Swimming behavior of *Aedes aegypti* larvae, when released in aqueous solution are illustrated in Fig. 2. It was observed that larval average swimming speed from control (Fig. 2A) was different from treated larvae (Fig. 2B). Immediately after contact, pyriproxyfen reduced the displacement of larvae and increased resting time with difference in number of meanders and angular velocity from the control.

**Figure 3:**
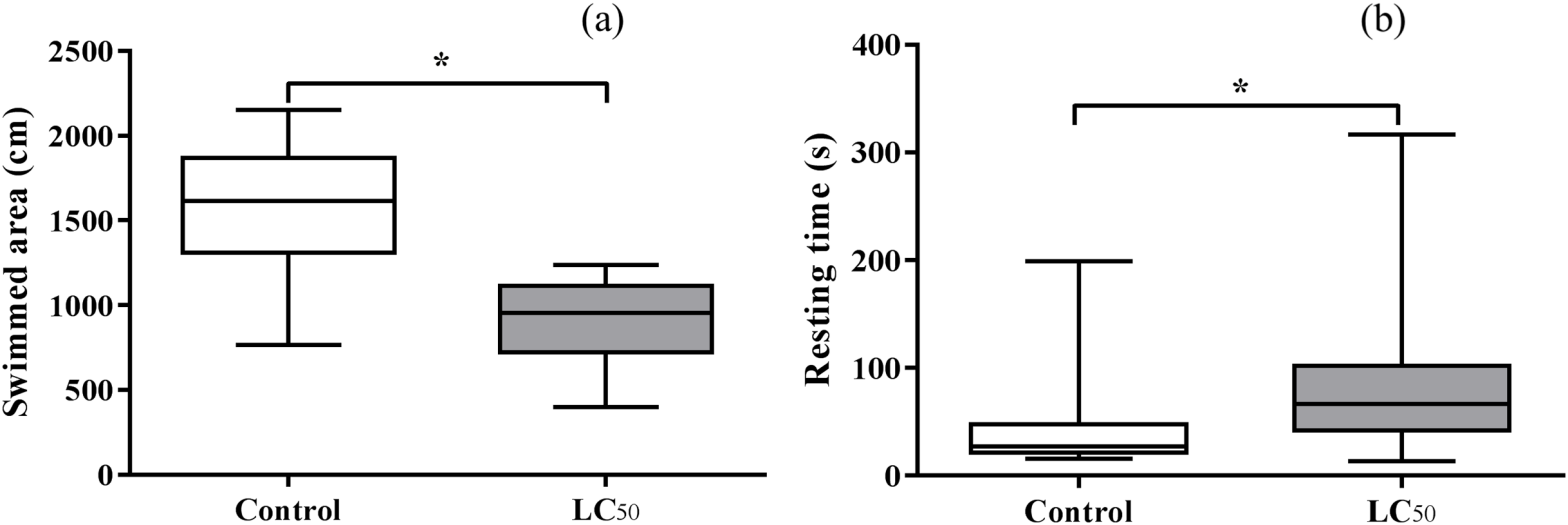
Means ± SD of swimmed area (A) and resting time (B) of third instar *Aedes aegypti* larvae exposed in aqueous solution for 24 h to LC_50_ pyriproxyfen concentrations. Bars followed by different letters differ at *P* < 0.05 (Tukey’s mean separation test). The bars represent mean values and the error bars are standard errors of the mean, asterisks (*) indicate significant differences between treatments.

Significant difference was observed between distance traveled in pyriproxyfen exposure LC_50_ aqueous solution and control (*t* = 6.40, d*f* = 1.32, *P* < 0.05). Distance traveled in control was higher than pyriproxyfen exposure LC_50_ aqueous solution (Fig. 3A). An increase in larval resting time (*t* = 2.09, d*f* = 1.38, *P* < 0.05) was found for those exposed to pyriproxyfen (Fig. 3B).

### Morphological analysis of the midgut

**Figure 4:**
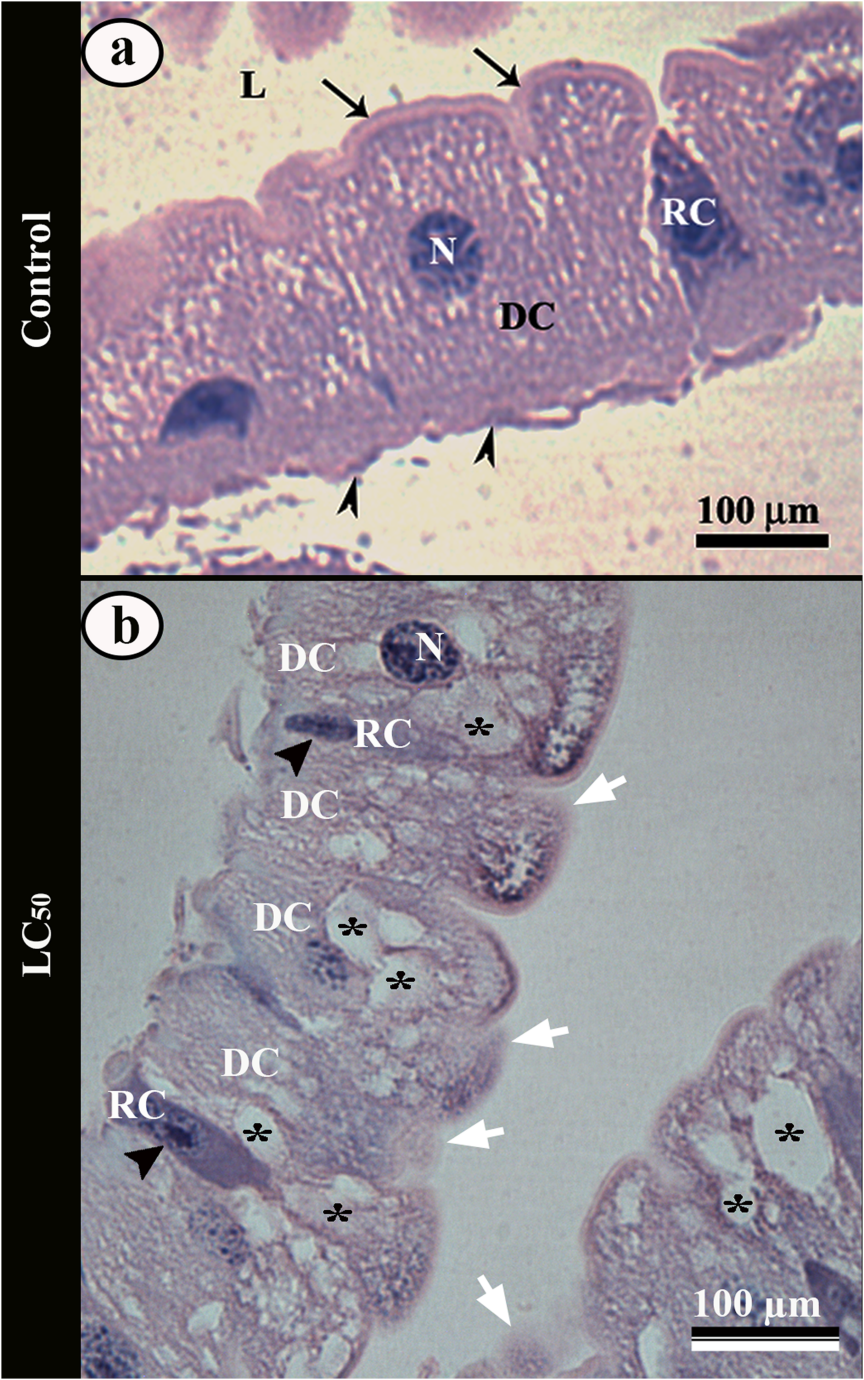
Light micrographs of the midgut of third instar *Aedes aegypti* larva. (A) Single layered epithelium with columnar digestive cells (DC) from control larvae, with spherical nucleus (N), well-developed brush border (arrows) and basal membrane (arrowheads). Note a regenerative cell (RC) in differentiation. (B) Midgut epithelium of larvae exposed to aqueous solution of LC_50_ pyriproxyfen showing digestive cells (DC) with cytoplasmic vacuoles (asterisks) and disorganized brush border (arrows). Regenerative cells (RC) from the larvae exposed in concentrations. Note many regenerative cells (RC) in differentiation with large nucleus (black arrow head). L – lumen.

The midgut of control *Aedes aegypti* larvae had a single layered columnar epithelium with median spherical nucleus and well-developed apical brush border (Fig. 4A). Some regenerative cells were in differentiation process for digestive ones, characterized by a basophil cytoplasm (Fig. 4A).

The ingestion of pyriproxyfen caused disorganization of the midgut epithelium in the larvae, with increase in the number of cytoplasmic vacuoles, clear areas and disorganized brush border (Fig. 4B). In these larvae there was an increase in the amount of regenerative cells differentiation characterized by many regenerative cells with large nucleus (Fig. 4B).

**Figure 5:**
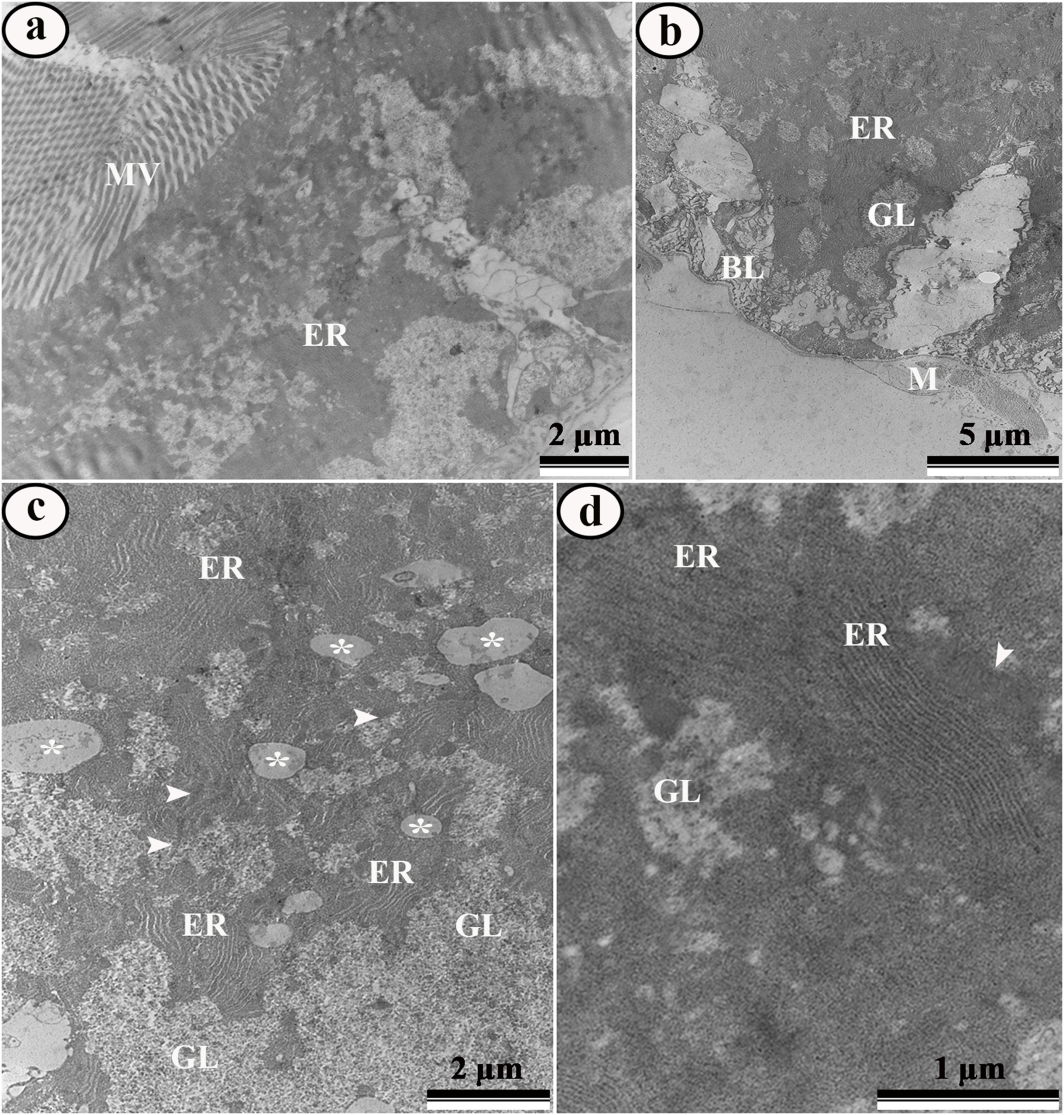
Transmission electron micrographs of the digestive cells from midgut of control third instar *Aedes aegypti* larvae. (A) General view of digestive cell, showing microvilli (MV) and rough endoplasmic reticulum (ER). (B) Basal region of digestive cell showing rough endoplasmic reticulum (ER), glycogen island (GL), regular basal labyrinth and muscle (M). (C) Perinuclear cytoplasm showing lipid droplets (asterisks), rough endoplasmic reticulum (ER), glycogen (GL), and mitochondria (arrowhead). (D) Detail of rough endoplasmic reticulum (ER), mitochondria (arrowhead) and glycogen (GL).

Ultrastructural analysis of the midgut cells in *Aedes aegypti* larvae from control showed digestive cells with many apical microvilli and rough endoplasmic reticulum (Fig. 5A) and basal plasma membrane with infoldings (Fig. 5B). Perinuclear cytoplasm was rich in lipid droplets, glycogen (Fig. 5C) mitochondria and rough endoplasmic reticulum (Figs. 5C–5D).

**Figure 6:**
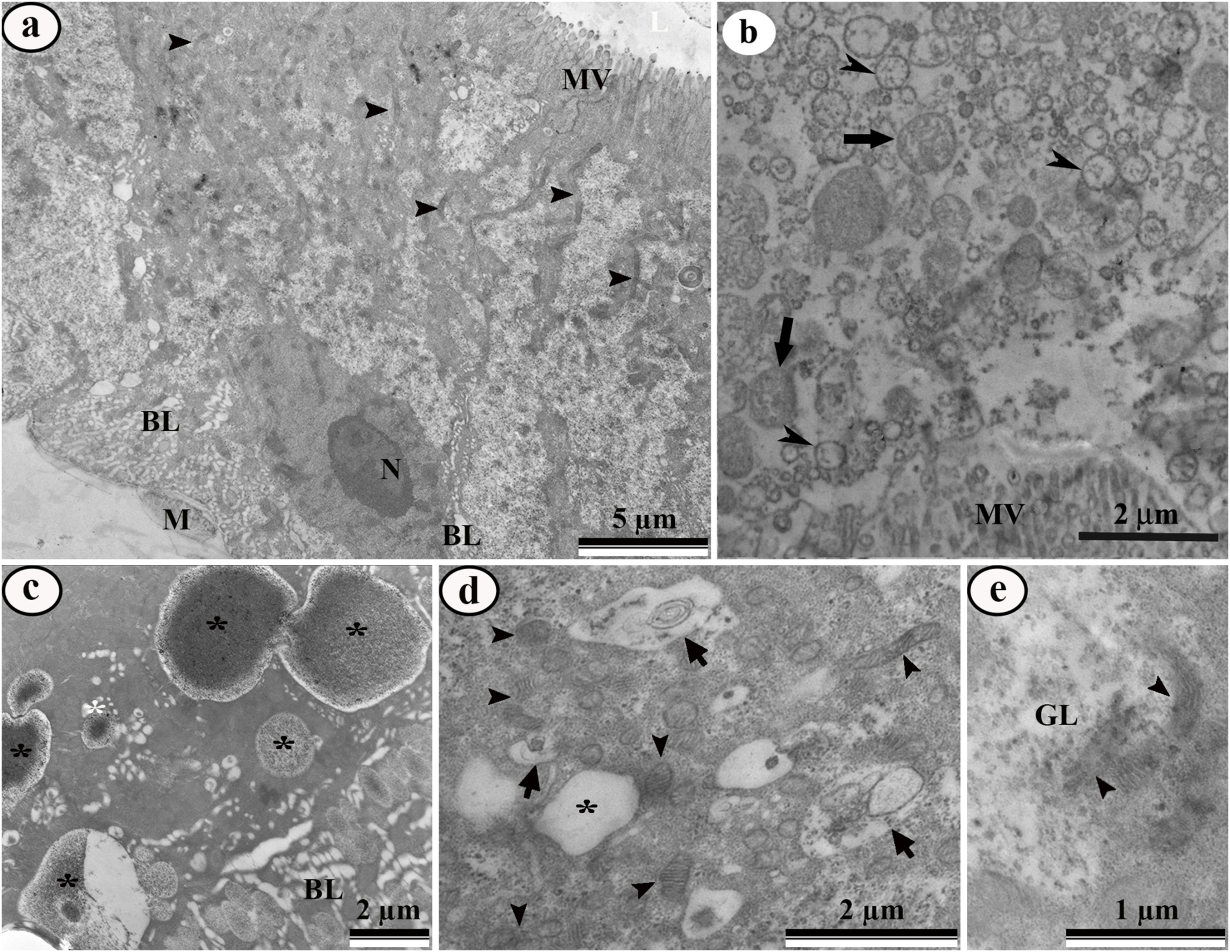
Transmission electron micrographs of the digestive cells from midgut of third instar *Aedes aegypti* larvae exposed to LC_50_ pyriproxyfen aqueous solution. (A) General view showing damaged microvilli (MV), Nucleus (N), mitochondria (arrowhead) and enlarged basal labyrinth (BL) and muscle (M). (B) Midgut lumen showing cell debris similar to mitochondria (arrows) and rough endoplasmic reticulum (arrowheads). (C) Basal cell region showing big lipid droplets (asterisks) and enlarged basal labyrinth (BL). (D) Perinuclear cytoplasm with autophagic vacuoles (arrows), lipid droplet (asterisk) and damaged mitochondria (arrowheads). (E) Details of damaged mitochondria (arrowheads) and empty glycogen deposit (GL).

**Figure 7:**
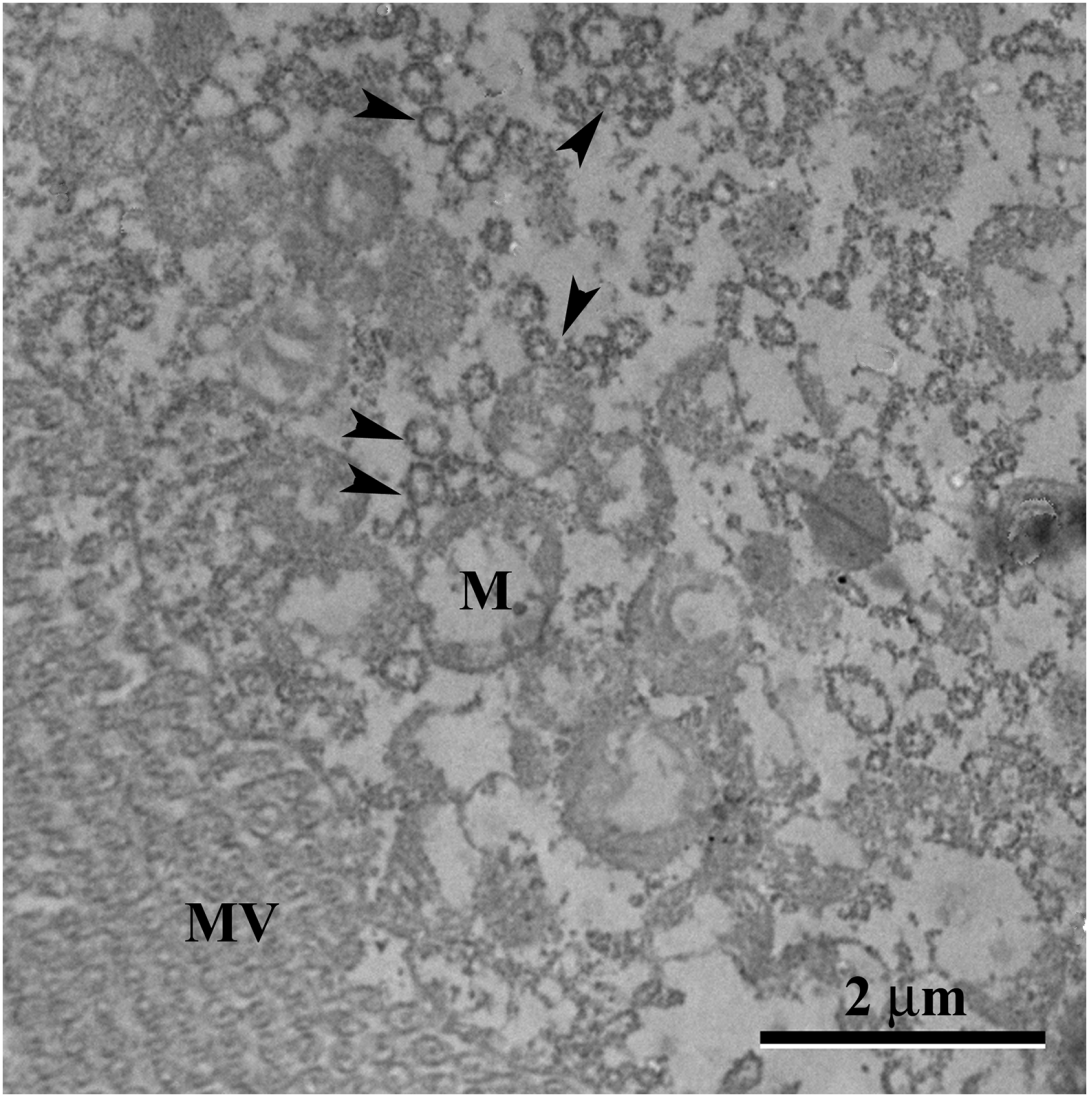
Transmission electron micrograph of the digestive cell form midgut of third instar *Aedes aegypti* larvae exposed to LC_50_ pyriproxyfen aqueous solution. Apical region showing damaged mitochondria (M) and vesicular rough endoplasmic reticulum (arrowheads). MV – microvilli.

*Aedes aegypti* larvae exposed to LC_50_ pyriproxyfen aqueous solution represented damaged midgut digestive cells, including microvilli fragmentation and cytoplasm disorganization characterized by extensive electron-lucent areas (Fig. 6A). The midgut lumen had plenty of cell debris (Fig. 6B). The basal cytoplasm was rich in enlarged lipid droplets, some of them coalescing to form big droplets (Figs. 6C–6D). The basal labyrinth had enlarged extracellular space (Fig. 6C). The cytoplasm had many deformed mitochondria (Figs. 6D–6E), autophagic vacuoles (Fig. 6D), almost empty glycogen deposits (Figs. 6E and 7), and depletion of endoplasmic reticulum characterized by change of flattened to vesicular cisternae (Fig. 7).

### Immunofluorescence

**Figure 8:**
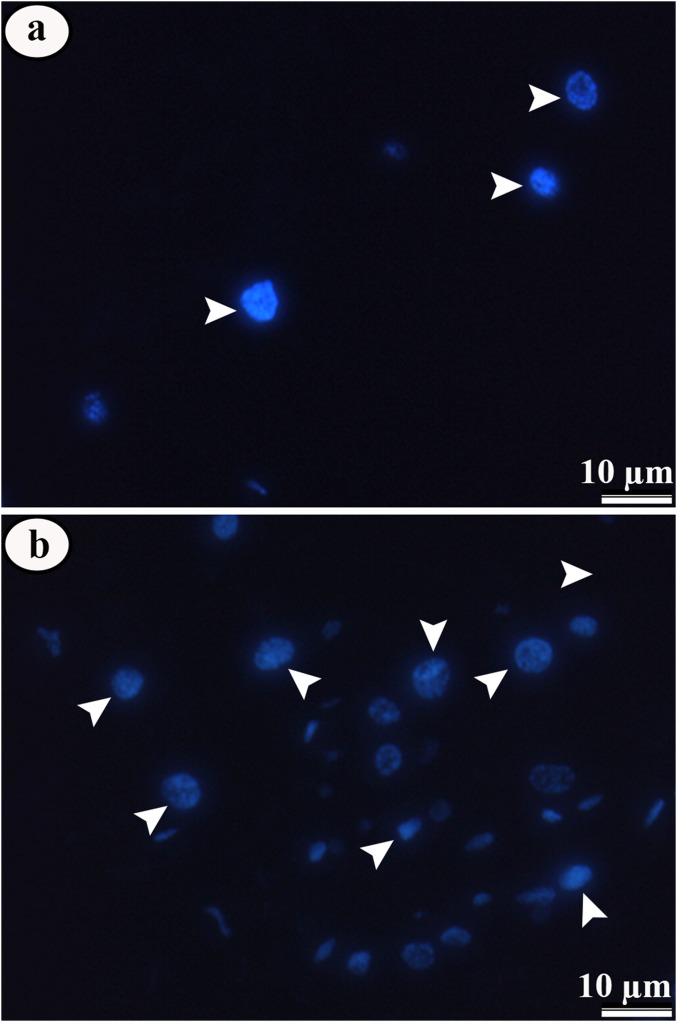
Micrographs of midgut epithelium of third instar *Aedes aegypti* larvae in the control and exposed to LC_50_ pyriproxyfen. Micrographs of midgut epithelium of third instar *Aedes aegypti* larvae in the control (A) and exposed to LC_50_ pyriproxyfen (B) showing negative staining for phosphorylate histone-H3, but with increase in the number of cell nucleus (blue) in treated ones.

The immunofluorescence to identify cell proliferation showed negative results for phosphorylate histone-H3 in both control and treated larvae, but there was an increase in the number of cell nucleus DAPI-stained in larvae exposed to pyriproxyfen (Figs. 8A–8B).

## Discussion

Our toxicological findings strongly suggest that pyriproxyfen is toxic to *Aedes aegypti* larvae supporting the potential use of this compound for the control this insect vector. Similar level of efficacy against mosquito larvae have been found for monoterpenes (Silva et al., 2018), squamocin (Costa et al., 2014) and essential oils combined with permethrin (Gross et al., 2017).

The pyriproxyfen efficacy against *Aedes aegypti* larvae is proved by the concentration killing 99% of larvae almost equal to other bioinsecticides like squamocin (Costa et al., 2014), essential oils (Aguiar et al., 2015), and imidazolium salts (Goellner et al., 2018). In addition to have lower lethal doses as compared to other insecticides, pyriproxyfen is found to be suitable insecticide for autodissemination (Suman et al., 2018; Tuten et al., 2016) and its larvicide effects remains up to 8 months in field (Oo et al., 2018).

Significant changes in swimming behavior, including displacement and speed, found here is the evidence of sublethal effect of pyriproxyfen to *Aedes aegypti* larvae. Some insecticides have tendency to modify behavioral responses in insects as soon as toxic compound is detected on the body (Barson, Fleming & Allan, 1992; Watson & Barson, 1996). Verheggen et al. (2007) proved that plant volatiles and their constituents effectively disrupt the recognition process of the host substrate also influence the walking behavior of insect. Similar swimming behavior was found for *Aedes aegypti* exposed in aqueous solution of monoterpene bioinsecticides (Silva et al., 2018) and deltamethrin (Marriel et al., 2016).

Life history attributes, such as behavior, morphology and physiology may represent the adaptations to deal with predators. Highlighting these behaviors, swimming speed allows them in avoiding detection by predators (Engström-Öst & Lehtiniemi, 2004) and competing with feed sources (Alvarez Costa et al., 2018; Janssens & Stoks, 2012). In our experiment, pyriproxyfen acted quickly, compromising larval displacement and ultimately making them detectible to predators in natural conditions. The relationship of this behavior generated by sublethal concentrations of pesticides is already observed in *Aedes aegypti* and *Anopheles pseudopunctipennis* (Diptera: Culicidae) larvae treated with temephos, permethrin, and *Eucalyptus nitens* essential oil (Alvarez Costa et al., 2018).

Although midgut is not a target organ to pyriproxyfen it is the first region that molecule interact and needs cross to be widespread into the hemocoel. To date, how insecticide molecules overcome the midgut epithelium barrier is poorly understood and it is an important subject matter. Here, we show that pyriproxyfen is not only transported from the midgut lumen to the hemolymph, because it causes histological changes in digestive cells of *Aedes aegypti* larvae characterizing brush border disorganization, intense cytoplasmic vacuolization and differentiation of regenerative cells. Vacuolization and damage to brush border suggests that digestive cells are dying, due to the insecticide toxicity (Costa et al., 2014; Hazarika et al., 2018; Vasantha-Srinivasan et al., 2018). The same histopathological effect was observed in midgut cells of *Aedes aegypti* (Gaban et al., 2015; Costa et al., 2014) and other insects when treated with different bio and chemical insecticide (Fiaz et al., 2018a, 2018b; Martínez et al., 2018a, 2018b).

Ultrastructural analyses show that pyriproxyfen had a cytotoxic effect on midgut cells of *Aedes aegypti* larvae. Midgut of treated larvae has damaged epithelial layer, with cell debris released in the midgut lumen, similar to found in this insect exposed to other new chemistry insecticides (Gaban et al., 2015). Cytoplasm vacuolization followed by cell breakdown releasing cell debris into the midgut lumen has been associated with digestive cell death caused by xenobiotics in different insects (Costa et al., 2014; Fiaz et al., 2018a; Suresh Kumar et al., 2013; Mangia et al., 2018; Martínez et al., 2018b).

Presence of abundant lipid droplets in treated midgut of *Aedes aegypti* with pyriproxyfen in our experiment, is consistent with previous findings (Valzania et al., 2018). Neutral lipid droplets contain triacylglycerols and cholesteryl esters which provide great energy reserves to the cell (Brasaemle, 2007). Lipid droplets are exploited during cell stresses (Henne, Reese & Goodman, 2018) releasing energy in response to the demands of insects (Arrese & Soulages, 2010) as well signaling for immune response (Cheon et al., 2006; Dettloff et al., 2001; Mullen & Goldsworthy, 2003). Glycogen and lipids both are energy reservoirs, however, glycogen is stored in polymers, readily degraded on demand to be used as glycolytic fuel (Steele, 1982), whereas lipids are used as energy source through β-oxidation (Athenstaedt & Daum, 2006).

The increase in the occurrence of autophagosomes in midgut digestive cells exposed by pyriproxyfen indicates that these cells may undergo cytoplasm reorganization, since autophagy is a constitutive process of cell compounds turnover (Nagata, 2018) and can be triggered in response to overcome energy depletion, oxidative stress, organelles damage, hypoxia or DNA damage (Kroemer, Mariño & Levine, 2010). Similar effects occur in the midgut cells of insects exposed to other toxic molecules (Costa et al., 2014; Fiaz et al., 2018b; Martínez et al., 2018a).

Mitochondrial deformation in the midgut cells found in this study is an indication of pyriproxyfen toxicity to *Aedes aegypti*. Mitochondrial functions are linked to their morphology and membrane ultrastructure (Vincent et al., 2016), and deformation may result in mitochondrial disfunction leading to cell death (Fiaz et al., 2018b). Similar features occur in the midgut of *Anitcarsia gemmatalis* (Lepidoptera: Noctuidae) exposed to squamocin (Fiaz et al., 2018a) and tebufenozide (Fiaz et al., 2018b).

An intriguing finding in *Aedes aegypti* larvae exposed to pyriproxyfen is depletion of rough endoplasmic reticulum fragmented in to small vesicles, suggesting that main function of midgut digestive cells in synthesizing protein (Fialho et al., 2009) almost cease. Perhaps much of energy expended for protein synthesis (Alberts et al., 2014) may be used for cell detoxification. In fact, detoxificant cytochrome P450 monooxygenases have been found in the midgut cells of insects exposed to xenobiotics (Liu et al., 2018). In addition, pyriproxyfen operates at molecular level by altering or inhibiting protein translation (Nisbet, 2000).

Since the pyriproxyfen causes damages in the midgut digestive cells, would be expected that these should be replaced by regenerative ones in response to infections (Buchon et al., 2009) and xenobiotics (Forkpah et al., 2014). Surprisingly, we did not detect proliferation in the midgut regenerative cells by immunofluorescence with anti-phospho-histone H3, an antibody that recognizes cell proliferation in the midgut of *Aedes aegypti* larvae (Fernandes et al., 2014, 2015). This finding indicates that *Aedes aegypti* larvae may not trigger the cell proliferation program after exposed to pyriproxyfen. Similar results are reported in *Spodoptera frugiperda* (Lepidoptera: Noctuidae) with Azadirachtin (Nisbet, 2000), cultured invertebrate and vertebrate cells (Salehzadeh et al., 2002) and cultured mosquito cells *Aedes albopictus* C6/36 (Diptera: Culicidae) with 20-hydroxyecdysone and non-steroidal ecdysone agonist (Smagghe et al., 2003). However, we find that the midgut of treated larvae has more digestive cells, as evidenced by increase in the amount of DAPI-labeled nucleus, suggesting that although regenerative cells did not undergo mitoses, they are in differentiation process to replace damaged digestive cells. The regenerative cells occur in nests with six to eight undifferentiated cells scattered along the midgut epithelium (Nardi, Bee & Miller, 2010; Nardi & Bee, 2012) and in some insects they are claimed to replace other midgut cells by differentiation other than proliferation (Martins et al., 2006; Rost-Roszkowska et al., 2010; Rocha et al., 2014).

## Conclusions

Overall, the present study leads to a useful strategy in contribution to the development of control strategies against *Aedes aegypti*. Despite being a juvenile hormone analog, pyriproxyfen acted quickly provoking toxicity not only limited to behavioral changes, but also elicited morphological damage to midgut cells. Thus, further investigations aiming to evaluate other non-target organs of this chemical would contribute to a better understanding of the potential of pyriproxyfen for an insect control program.

## Additional Information and Declarations

### Data Availability

The following information was supplied regarding data availability: The raw data is available at Figshare: Fiaz, Muhammad (2019): Fig 1 S1.xlsx. figshare. Dataset. https://doi.org/10.6084/m9.figshare.7462082.v1. Fiaz, Muhammad (2019): Fig 3 S2.xlsx. figshare. Dataset. https://doi.org/10.6084/m9.figshare.7462094.v1.
